# Supplementary material for: Applying Tissue Slice Culture in Cancer Research—Insights from Preclinical Proton Radiotherapy
Source: Cancers (Basel). 2020 Jun 16;12(6):1589. doi: 10.3390/cancers12061589 (PMC7352770; doi:10.3390/cancers12061589)
Supplement: Supplementary file 1 [file cancers-12-01589-s001.zip › Supplementary_final/Supplementary Materials.pdf]

Supplementary Materials: Applying Tissue Slice Culture in Cancer Research—Insights from Preclinical Proton Radiotherapy

Theresa Suckert, Treewut Rassamegevanon, Johannes Müller, Antje Dietrich, Antonia Graja, Michael Reiche, Steffen Löck, Mechthild Krause, Elke Beyreuther and Cläre von Neubeck

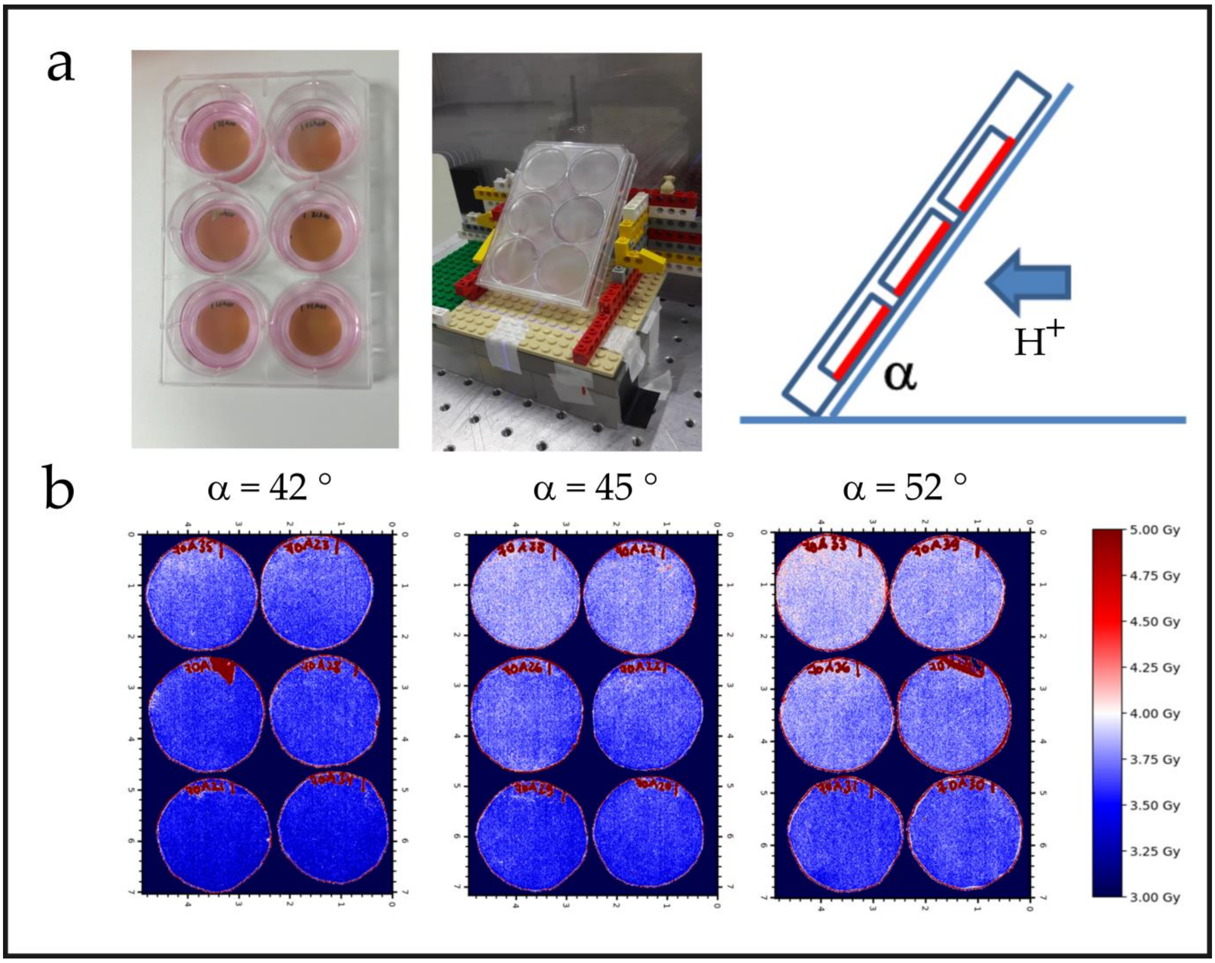

Figure S1. EBT3 measurements within the membrane inserts to evaluate dose homogeneity. (**a**) EBT3 films were placed into membrane inserts in 6-well plates with medium, using different volumes (Table S1). The plates were irradiated at different angles. The proton beam was delivered through the plate bottom. (**b**) Homogeneous dose distributions were observed within the individual inserts independent of medium volumes. However, the vertical plate orientation and the related distance to the proton beam (inverse square law) induced effects on the dose distribution across the plate.

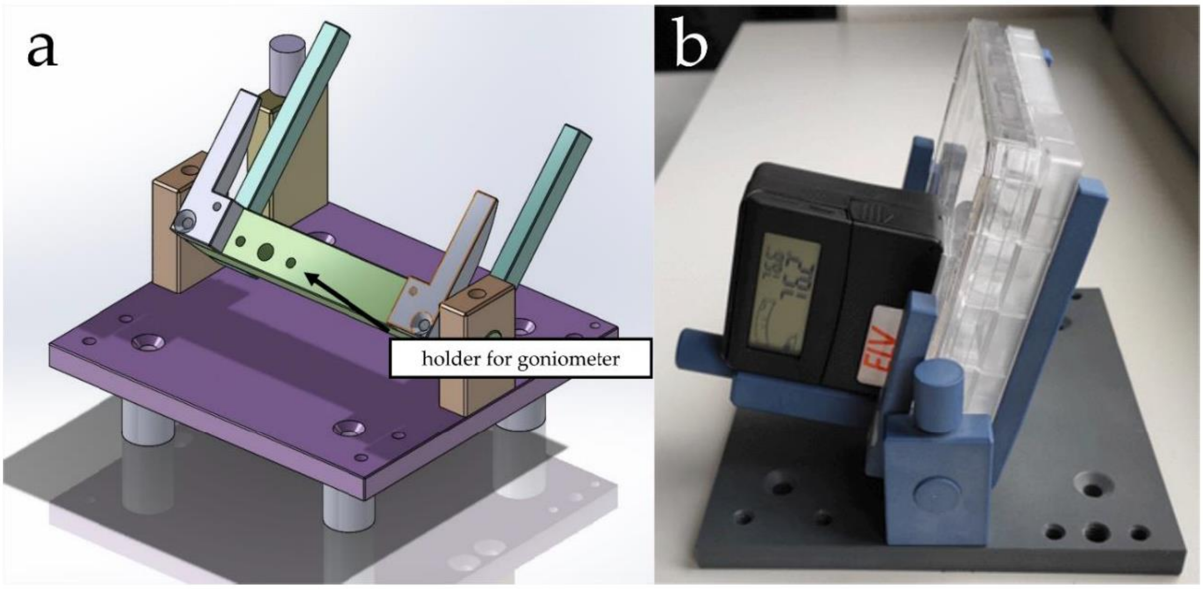

Figure S2. (**a**) Mechanical drawing and (**b**) lateral view of the milled setup for proton irradiation of well plates. The device consists of acrylonitrile butadiene styrene and polymethylmethacrylate and is equipped with an adjustable automatic goniometer for accurate angle measurements. The blueprint is provided in the Supplementary File 1.

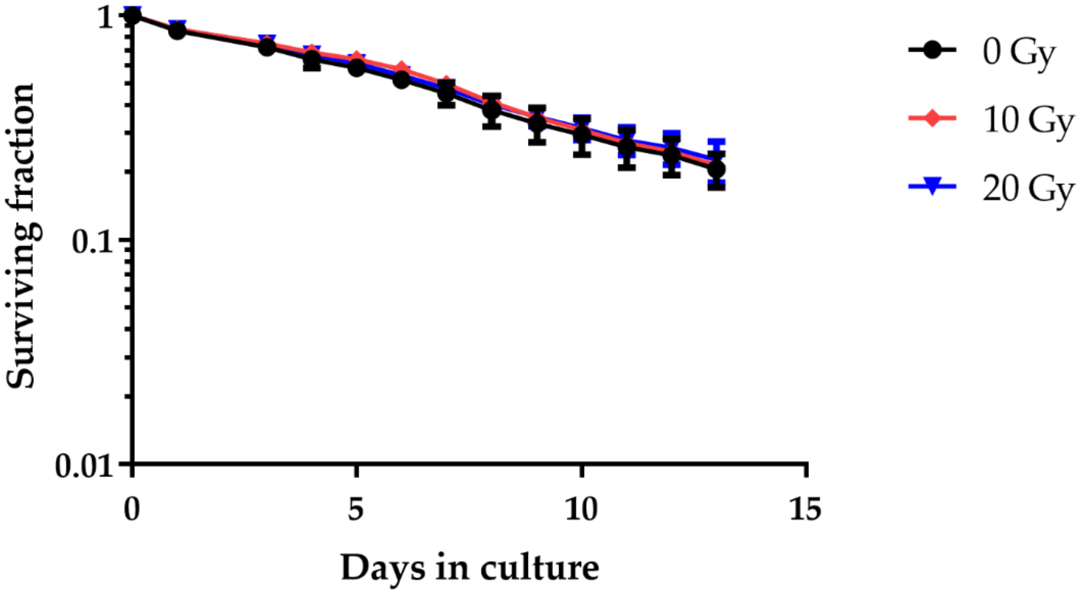

Figure S3. Cell survival of Cal33 tumor slices as measured via LDH release into the medium. Irradiation at day 4 did not induce an increased cell death. Both irradiated and non-irradiated tumor slices exhibited a high LDH release across the whole cultivation period (mean with SD, n = 3).

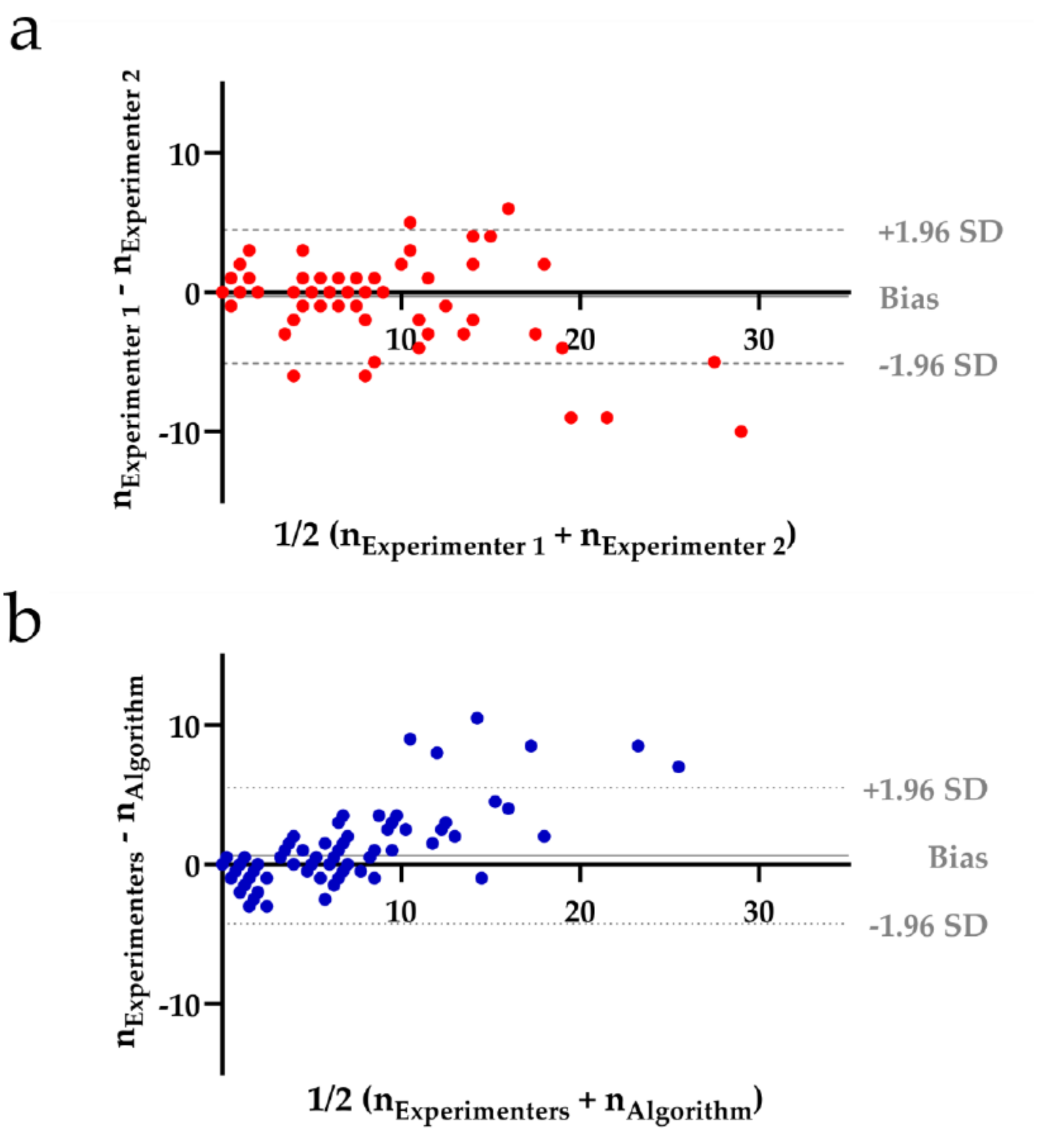

Figure S4. Bland-Altman analysis of foci counting methods. Plots compare the counted foci numbers of (**a**) the two independent observers (TR, TS) and (**b**) the average count of the observers to the algorithm. While both counting methods had a comparable standard deviation and the algorithm did not have a general bias towards higher or lower cell numbers, automated detection tended to miscount foci in cells with very low or high foci numbers.

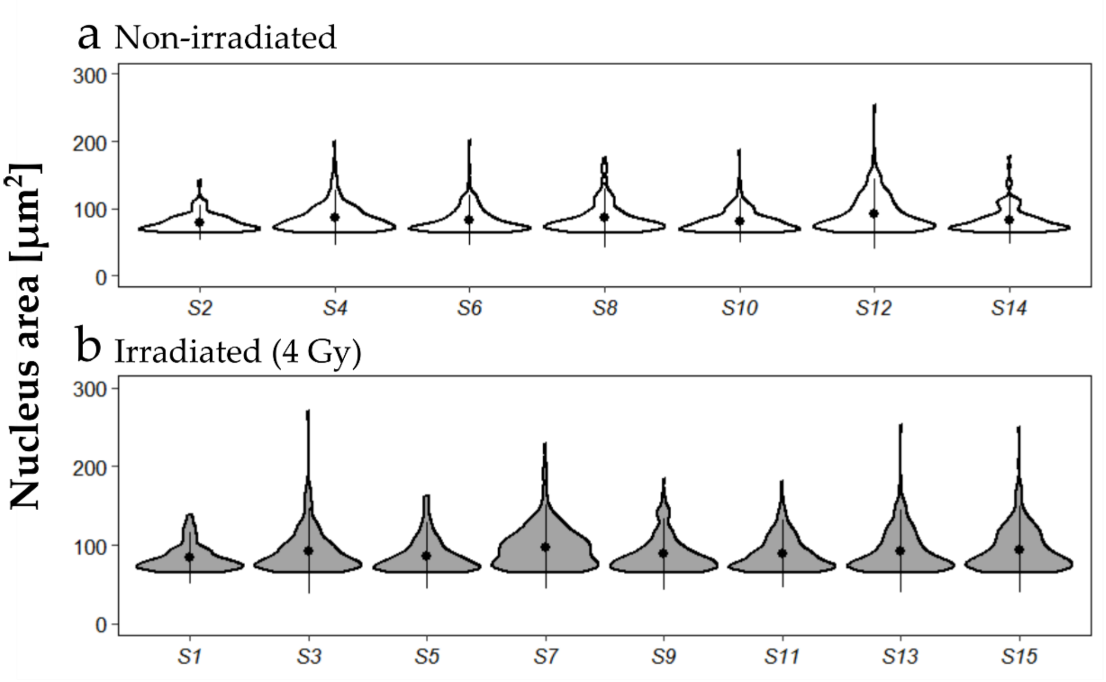

Figure S5. Nucleus area of (**a**) non-irradiated and (**b**) irradiated individual tumor slices determined by DAPI staining. A linear mixed-effects model was used to investigate data homogeneity [45]. There was no difference in nucleus area within the treated and untreated groups.

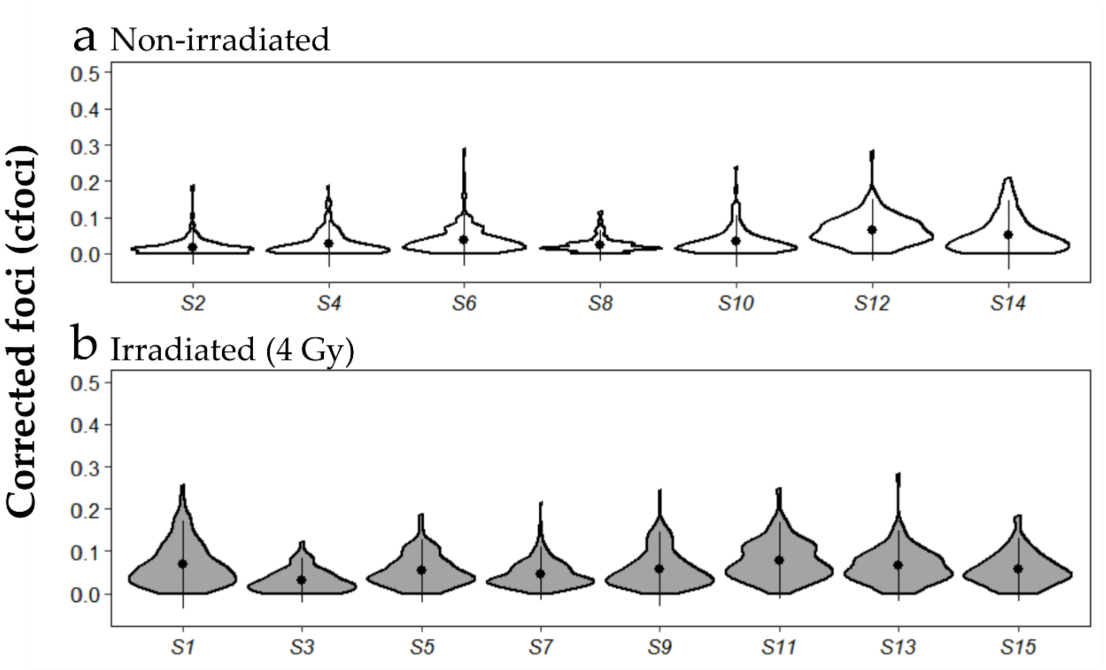

Figure S6. Corrected foci (cfoci) of individual (**a**) non-irradiated and (**b**) irradiated tumor slices (S). Slices were alternating allocated in the treatment groups. A linear mixed-effects model was applied to determine the heterogeneity of cfoci among tumor slices within the conditions [45]. Inside the treated- and untreated groups, no statistically significant difference was found.

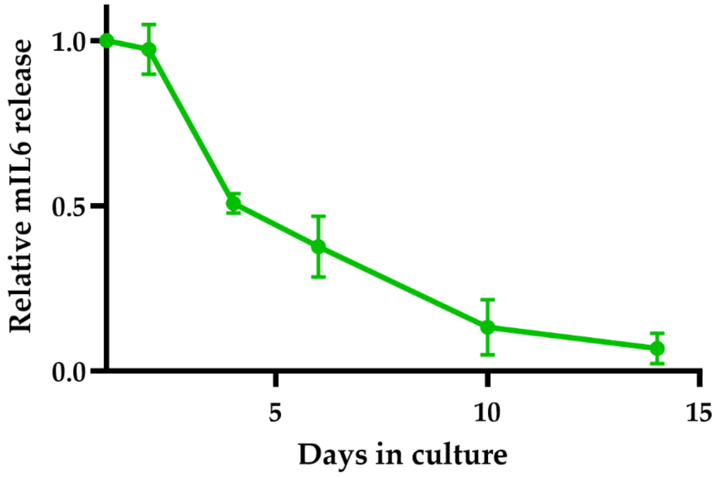

Figure S7. Release of the cytokine IL6 by OBSC into the supernatant relative to day 1 in culture. An intense inflammatory reaction was noted on the first day in culture (mean with SD = 1769 ± 203 pg/ml), which only decreased by day 4, postponing the start of the proton radiation experiments to this day (n = 12).

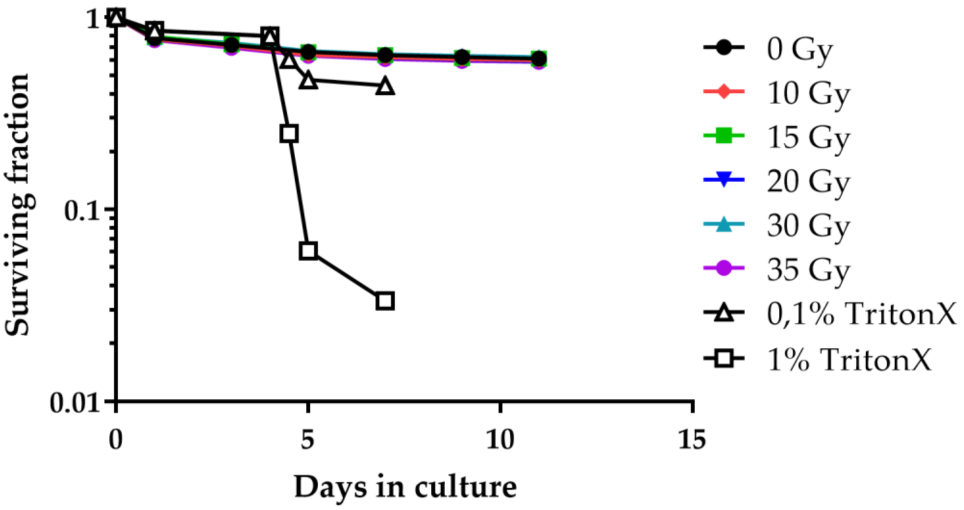

Figure S8. Cell survival of OBSC after proton irradiation with different doses. Slices (n = 6) were irradiated at day 4 in culture with doses ranging from 10 – 35 Gy and compared to non-irradiated controls (n = 30). No increased cell death could be measured with the LDH assay for any dose group at any time point. In contrast, cell death induction with TritonX (n = 3) showed a concentration-dependency.

Table S1. Dose values measured in the inserts at different beam angles. The distance to the proton beam in upper, middle, and lower rows affected the detected dose while the different volumes of medium had no influence.

**angle**	**42 °**		**45 °**		**52 °**	
Dose [Gy]	left column	right column	left column	right column	left column	right column
upper row	3.68	3.65	3.82	3.78	3.88	3.81
middle row	3.56	3.59	3.69	3.66	3.77	3.73
lower row	3.43	3.43	3.55	3.57	3.61	3.64
Medium vol.	1.2 ml	1 ml	1.2 ml	1.2 ml	1.2 ml	1.5 ml

Table S2. List of used primary and secondary antibodies. The specific combinations are indicated by the number # of the respective antibodies.

#	Primary antibody	Supplier	Cat.no.	Dilution	#2nd
1	mouse anti-Bromodeoxyuridine	Agilent[1]	M074401-8	1:50	1
2	mouse anti-CD44	Agilent[1]	M7082	1:400	2
3	rat anti-GFAP	Merck[2]	345860	1:1000	3
4	rabbit anti-γH2AX (OBSC)	Bethyl Laboratories[3]	IHC-00059	1:500	4
5	mouse anti-γH2AX (TSC)	Merck[2]	05-636	1:1000	5
6	rabbit anti-Iba1	Abcam[4]	Ab178846	1:2000	6
7	rabbit anti-Ki67	Abcam[4]	Ab833	1:100	7
8	chicken anti-MAP2	Abcam[4]	Ab5392	1:1000	8
9	guinea pig anti-NeuN	Merck[2]	ABN90P	1:1000	9
10	rabbit anti-Oligodendrocyte Specific Protein	Abcam[4]	Ab53041	1:1000	10
11	rabbit anti-Pimonidazole	Hypoxyprobe[5]	HP3	1:50	1

#	Secondary antibody	Supplier	Cat.no.	Dilution	#1st
1	Animal Research Kit/HRP	Agilent[1]	K3954	user guide	1, 11
2	EnVision Detection System, Peroxidase/DAB	Agilent[1]	K500711	user guide	2, 7
3	anti-rat Alexa488	Invitrogen[6]	A21208	1:500	3
4	Thyramid SuperBoost Kit anti-rabbit Alexa594	Thermo Fisher Scientific[7]	B40925	user guide	4
5	Thyramid SuperBoost Kit anti-mouse Alexa488	Thermo Fisher Scientific[7]	B40912	user guide	5
6	anti-rabbit Dylight650	Thermo Fisher Scientific[7]	SA5-10041	1:500	6
7	EnVision™+ System_HRP, Rabbit (DAB+)	Agilent[1]	K401111	user guide	7
8	anti-chicken Rhodamie Red	Dianova[8]	703295155	1:100	8
9	anti-guinea pig Cy5	Abcam[4]	Ab102372	1:250	9
10	anti-rabbit Alexa594	Invitrogen[6]	A11012	1:500	10

[1] Agilent, Santa Clara, USA; [2] Merck, Darmstadt, Germany; [3] Bethyl Laboratories, Montgomery, USA; [4] Abcam, Cambridge, Great Britain; [5] Hypoxyprobe, Burlington, USA; [6] Invitrogen, Carlsbad, USA; [7] Thermo Fisher Scientific, Waltham, USA; [8] Dianova, Hamburg, Germany

Table S3. Description of the image evaluation workflows for H&E, DAPI and CD44 stained slices using Fiji. All scripts were written with ImageJ macro language and can be found on Github (https://github.com/Theresa-S).

Image	Preprocessing	Thresholding	Measure
H&E 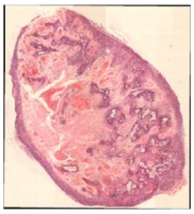	type = RGB Colour<br>manual background correction<br>Gaussian blur 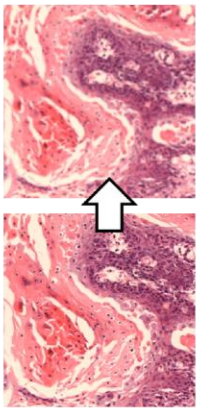<br>Colour deconvolution vectors = H&E 2<br>Channel1 (Viable area)<br>Channel2 (Whole tissue) 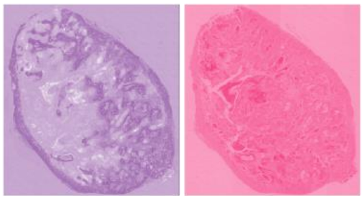	Method = Otsu<br>Make binary 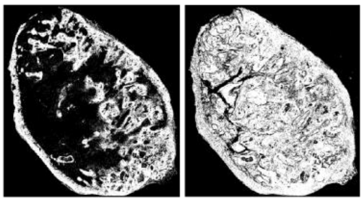	%area
DAPI 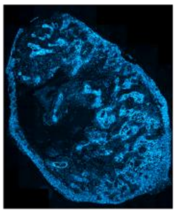	manual background correction<br>Manual masking of tumor	Method = Triangle dark<br>Make binary<br>Watershed 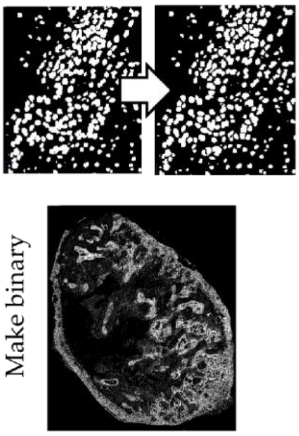	%area
CD44 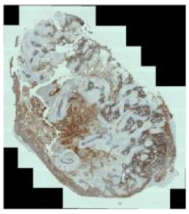	type = RGB Colour<br>duplicate<br>Duplicate 1: 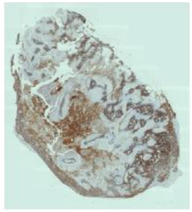<br>manual background correction<br>Colour deconvolution vectors = H DAB<br>Channel2 (CD44) 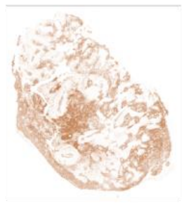	Duplicate 2:<br>Method = Triangle<br>Make binary<br>fill holes<br>convert to mask<br>Apply selection onto CD44 image 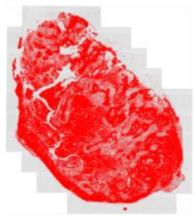	mean intensity
