# Supplementary material for: Applying Tissue Slice Culture in Cancer Research—Insights from Preclinical Proton Radiotherapy
Source: Cancers (Basel). 2020 Jun 16;12(6):1589. doi: 10.3390/cancers12061589 (PMC7352770; doi:10.3390/cancers12061589)
Supplement: Supplementary file 1 [file cancers-12-01589-s001.zip › Supplementary_final/Blueprint_setup [german].zip!/Blueprint_setup [german]/000 - Stückliste Kippmechanik.pdf]

POS-NR.	BENENNUNG	BESCHREIBUNG	MENGE
1	001 - Grundplatte		1
2	003 - Halter seitlich		2
3	004 - Kippvorrichtung Grundteil		1
4	006 - Kippvorrichtung Front rechts		1
5	007 - Kippvorrichtung Rückseite links		1
6	005 - Kippvorrichtung Front links		1
7	008 - Kippvorrichtung Rückseite rechts		1
8	002 - Distanz		4
9	010 - Halter Winkelmesser		1
10	012 - Arretierzapfen		2
11	011 - Zapfen		1
12	009 - Zapfen kurz		2
13	O-Ring, 12,00x1,00 mm, NBR (70A)		2
