# Supplementary material for: Applying Tissue Slice Culture in Cancer Research—Insights from Preclinical Proton Radiotherapy
Source: Cancers (Basel). 2020 Jun 16;12(6):1589. doi: 10.3390/cancers12061589 (PMC7352770; doi:10.3390/cancers12061589)
Supplement: Supplementary file 1 [file cancers-12-01589-s001.zip › Supplementary_final/Blueprint_setup [german].zip!/Blueprint_setup [german]/002 - Distanz.PDF]

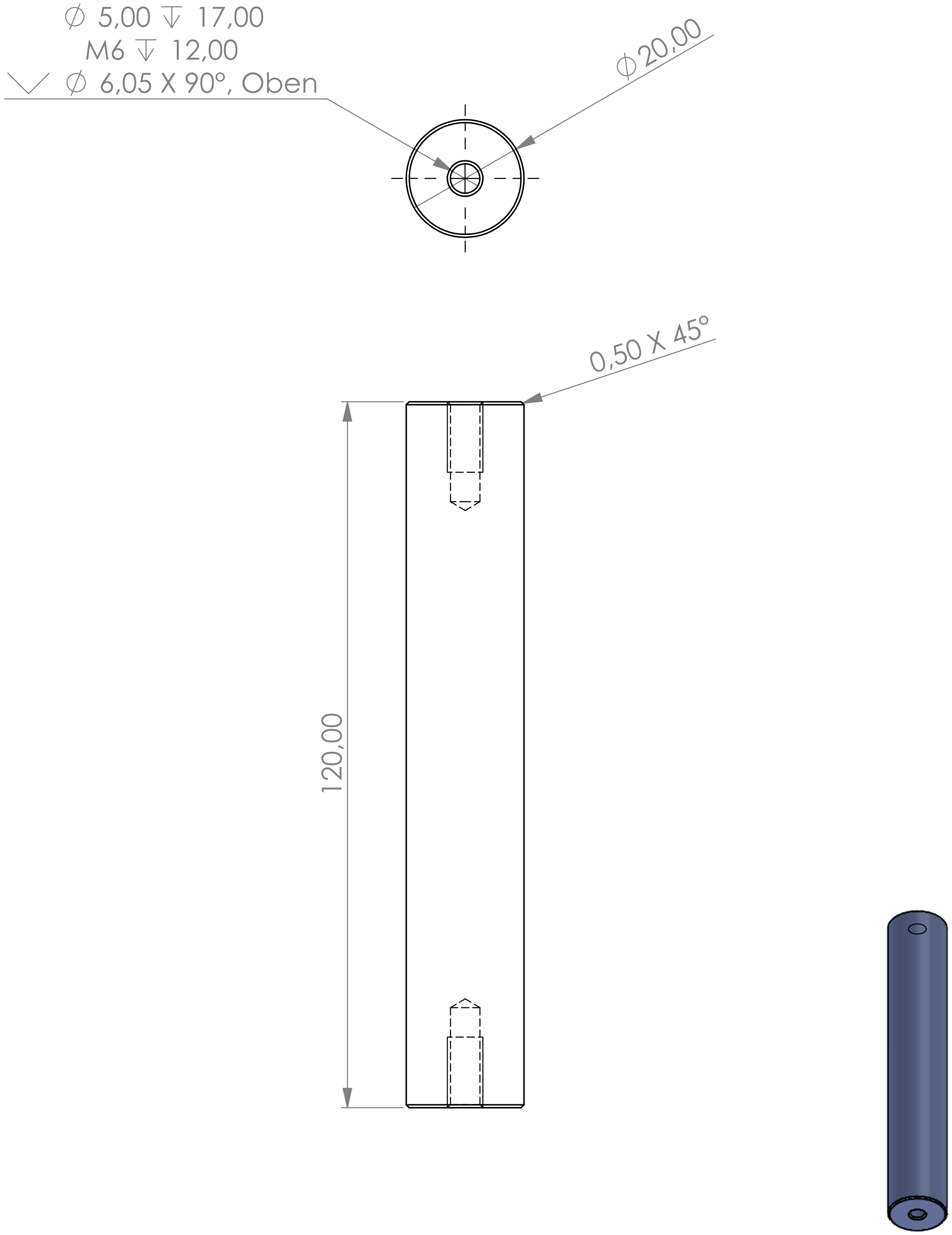

Oberfläche	Allgemeintoleranz	Werkstoff
geschlichtet	DIN ISO 2786-mk	SIKABLOCK M960

Erstellt von	Genehmigt von	Sachnummer
MReiche		Kippmechanik

Titel	Dokumentenart				
002 - Distanz					
	Maßstab	Änd.	Ausgabedatum	Spr.	Blatt
	1:1		18.03.2020		1/1
