# Supplementary material for: Applying Tissue Slice Culture in Cancer Research—Insights from Preclinical Proton Radiotherapy
Source: Cancers (Basel). 2020 Jun 16;12(6):1589. doi: 10.3390/cancers12061589 (PMC7352770; doi:10.3390/cancers12061589)
Supplement: Supplementary file 1 [file cancers-12-01589-s001.zip › Supplementary_final/Blueprint_setup [german].zip!/Blueprint_setup [german]/003 - Halter seitlich.PDF]

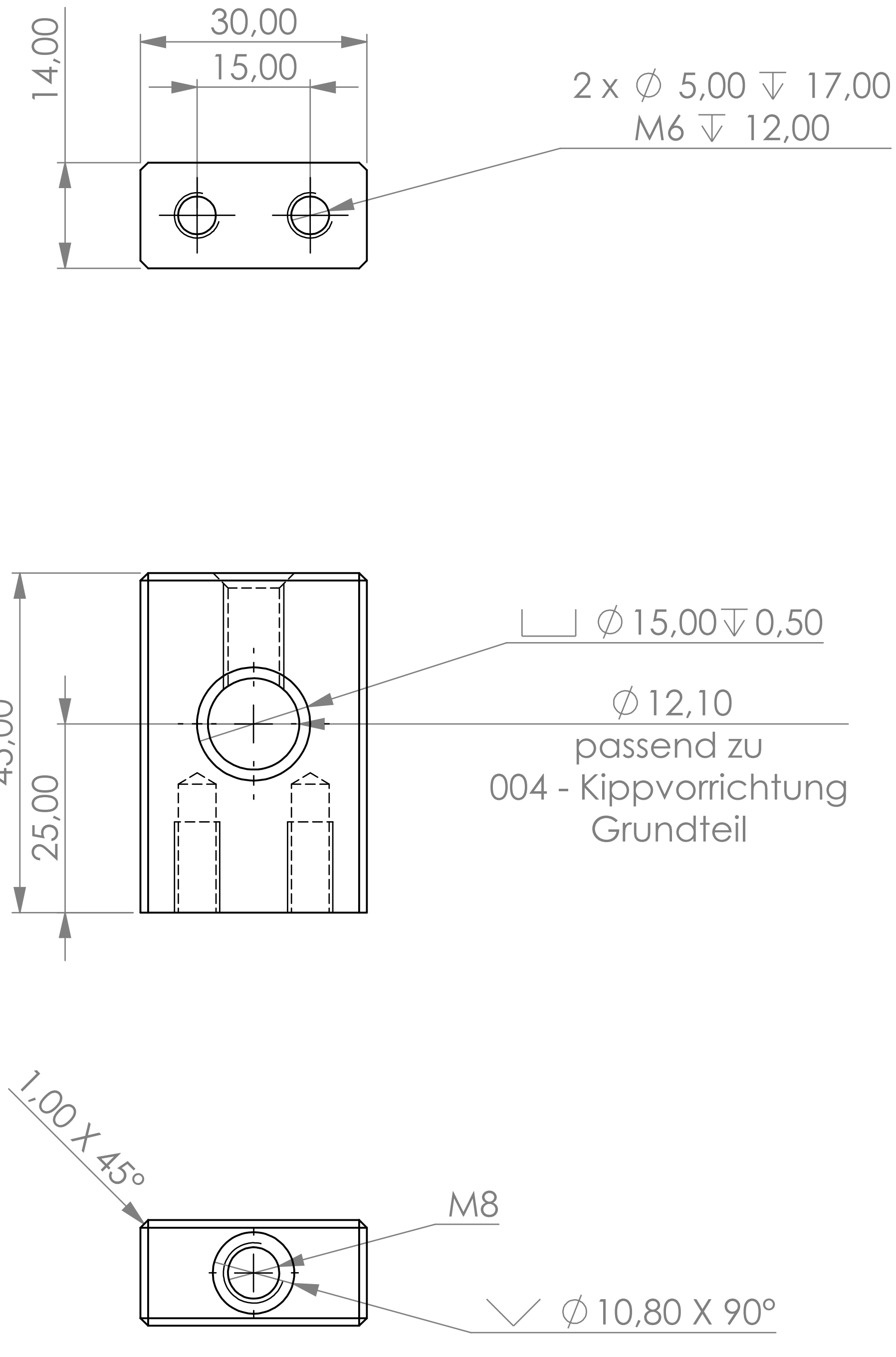

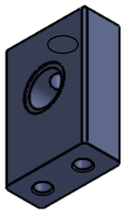

Oberfläche	Allgemeintoleranz			Werkstoff		
geschlichtet	DIN ISO 2786-mk			SIKABLOCK M960		
	Erstellt von: MReiche	Genehmigt von		Sachnummer: Kippvorrichtung		
	Titel: 003 - Halter seitlich	Dokumentenart				
		Maßstab: 1:1	Änd.	Ausgabedatum: 18.03.2020	Spr.	Blatt: 1/1
