# Supplementary material for: Applying Tissue Slice Culture in Cancer Research—Insights from Preclinical Proton Radiotherapy
Source: Cancers (Basel). 2020 Jun 16;12(6):1589. doi: 10.3390/cancers12061589 (PMC7352770; doi:10.3390/cancers12061589)
Supplement: Supplementary file 1 [file cancers-12-01589-s001.zip › Supplementary_final/Blueprint_setup [german].zip!/Blueprint_setup [german]/004 - Kippvorrichtung Grundteil.PDF]

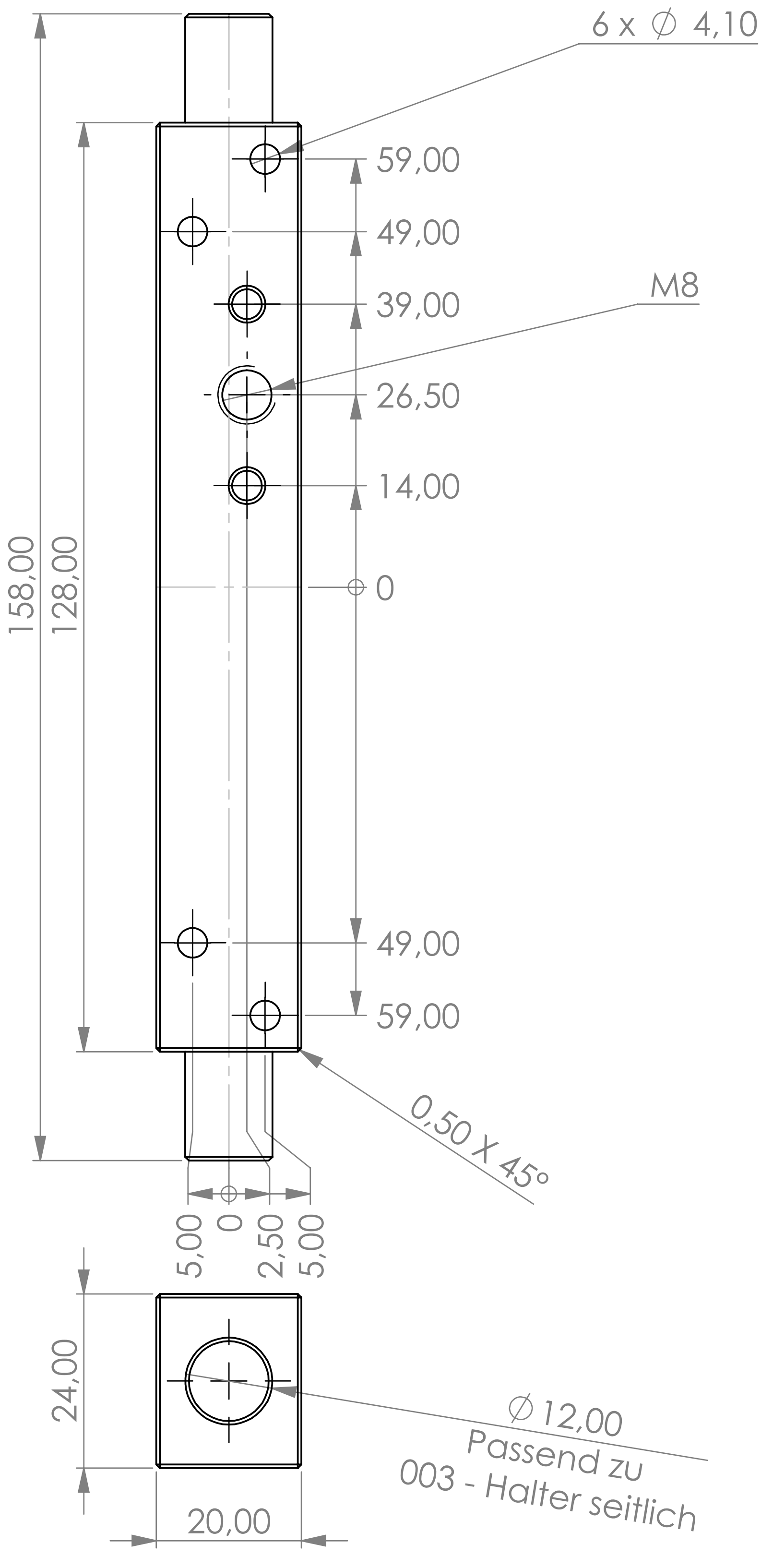

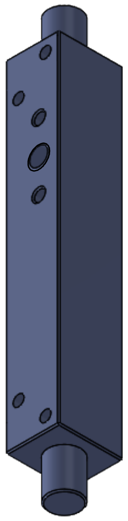

Oberfläche	Allgemeintoleranz			Werkstoff
geschlichtet	DIN ISO 2786-mk			SIKABLOCK M960
	Erstellt von: MReiche	Genehmigt von		Sachnummer: Kippmechanik
	Titel: 004 - Kippvorrichtung Grundteil	Dokumentenart		
		Maßstab: 1:1	Änd.	Ausgabedatum: 18.03.2020 / Spr. / Blatt: 1/1
