# Supplementary material for: Applying Tissue Slice Culture in Cancer Research—Insights from Preclinical Proton Radiotherapy
Source: Cancers (Basel). 2020 Jun 16;12(6):1589. doi: 10.3390/cancers12061589 (PMC7352770; doi:10.3390/cancers12061589)
Supplement: Supplementary file 1 [file cancers-12-01589-s001.zip › Supplementary_final/Blueprint_setup [german].zip!/Blueprint_setup [german]/007 - Kippvorrichtung Rückseite links.PDF]

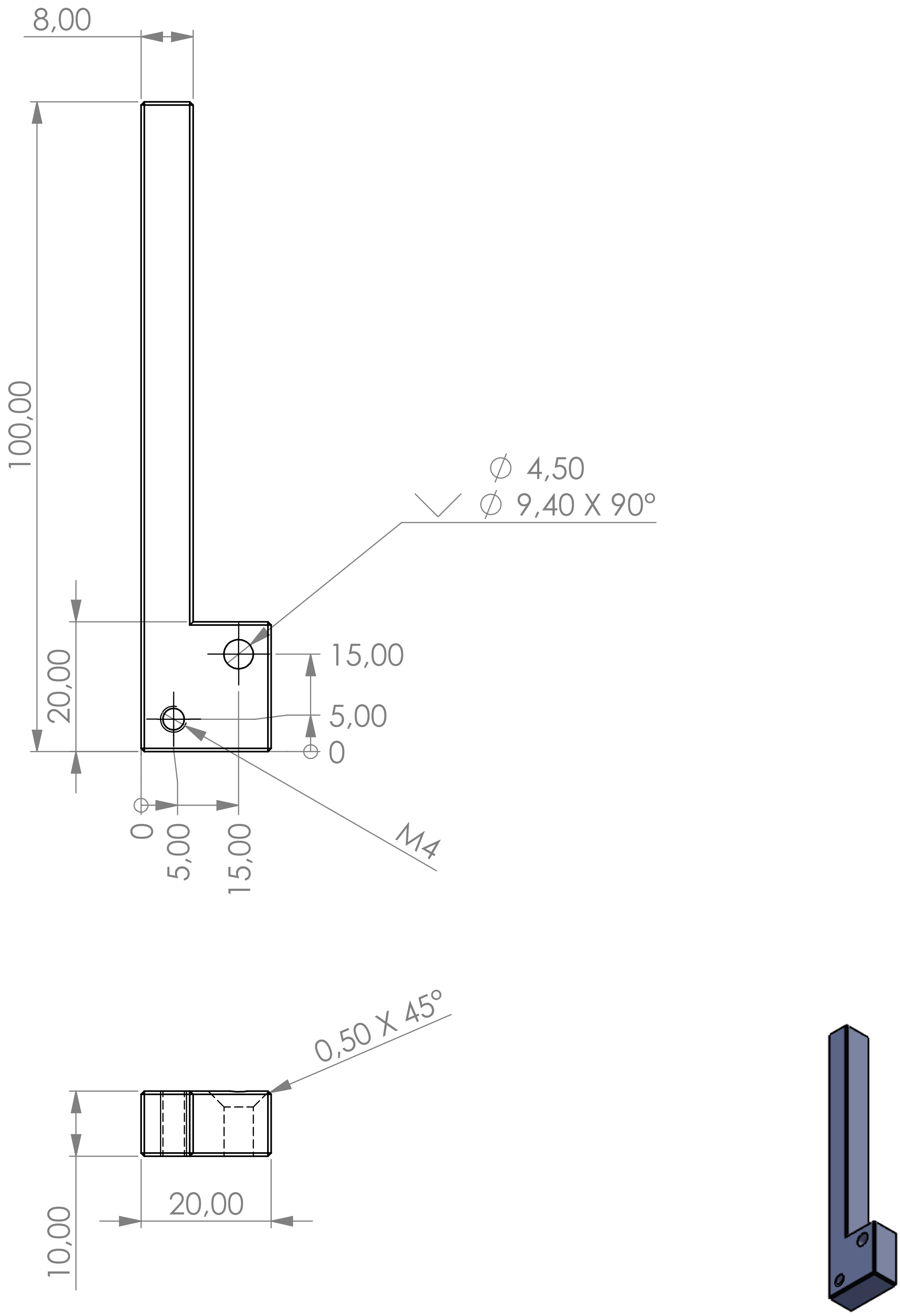

**Oberfläche**<br>geschlichtet	**Allgemeintoleranz**<br>DIN ISO 2786-mk	**Werkstoff**<br>SIKABLOCK M960
	**Erstellt von**<br>MReiche / **Genehmigt von**	**Sachnummer**<br>Kippmechanik
	**Titel**<br>007 - Kippvorrichtung Rückseite links	**Dokumentenart**

Maßstab	Änd.	Ausgabedatum	Spr.	Blatt
1:1		18.03.2020		1/1
