# Supplementary material for: Applying Tissue Slice Culture in Cancer Research—Insights from Preclinical Proton Radiotherapy
Source: Cancers (Basel). 2020 Jun 16;12(6):1589. doi: 10.3390/cancers12061589 (PMC7352770; doi:10.3390/cancers12061589)
Supplement: Supplementary file 1 [file cancers-12-01589-s001.zip › Supplementary_final/Blueprint_setup [german].zip!/Blueprint_setup [german]/009 - Zapfen kurz.PDF]

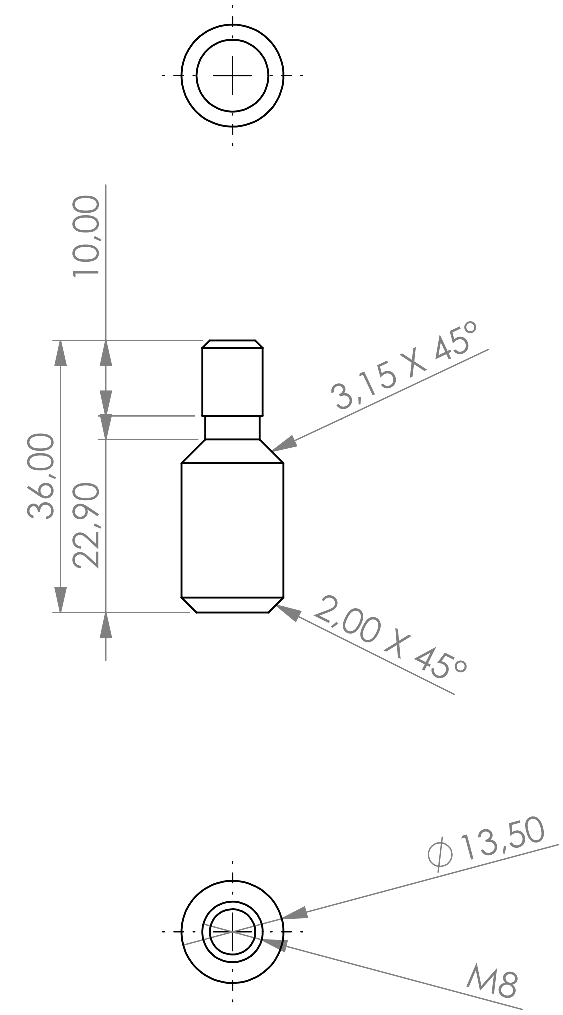

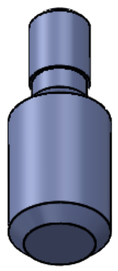

Oberfläche	Allgemeintoleranz		Werkstoff
geschlichtet	DIN ISO 2786-mk		SIKABLOCK M960
	Erstellt von: MReiche	Genehmigt von:	Sachnummer: Kippvorrichtung
	Titel: 009 - Zapfen kurz	Dokumentenart:	
		Maßstab: 1:1 / Änd.: / Ausgabedatum: 18.03.2020	Spr.: / Blatt: 1/1
