# Supplementary material for: Applying Tissue Slice Culture in Cancer Research—Insights from Preclinical Proton Radiotherapy
Source: Cancers (Basel). 2020 Jun 16;12(6):1589. doi: 10.3390/cancers12061589 (PMC7352770; doi:10.3390/cancers12061589)
Supplement: Supplementary file 1 [file cancers-12-01589-s001.zip › Supplementary_final/Blueprint_setup [german].zip!/Blueprint_setup [german]/012 - Arretierzapfen.PDF]

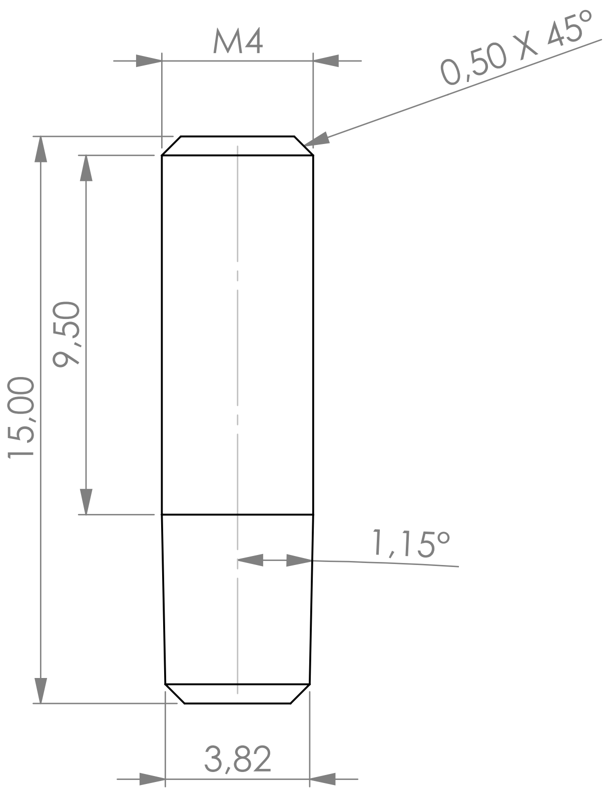

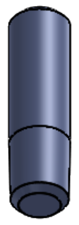

Oberfläche	Allgemeintoleranz	Werkstoff
geschlichtet	DIN ISO 2786-mk	SIKABLOCK M960

Erstellt von	Genehmigt von	Sachnummer
MReiche		Kippvorrichtung

Titel	Dokumentenart
012 - Arretierzapfen	

Maßstab	Änd.	Ausgabedatum	Spr.	Blatt
5:1		18.03.2020		1/1
